# Predictor Analysis in Radiofrequency Ablation of Benign Thyroid Nodules: A Single Center Experience

**DOI:** 10.3389/fendo.2021.638880

**Published:** 2021-05-17

**Authors:** Alessandro Bisceglia, Ruth Rossetto, Sara Garberoglio, Angelica Franzin, Alice Cerato, Francesca Maletta, Mauro Giulio Papotti, Ezio Ghigo, Loredana Pagano, Mauro Maccario, Roberto Garberoglio

**Affiliations:** ^1^ Division of Endocrinology, Diabetology and Metabolism, Department of Medical Sciences, University of Turin, Turin, Italy; ^2^ Centro Multidisciplinare Della Tiroide (CMT), Humanitas Cellini, Turin, Italy; ^3^ Pathology Unit, Department of Laboratory Medicine, City of Health and Science Hospital, Turin, Italy; ^4^ Pathology Unit, Department of Oncology, University of Turin and City of Health and Science Hospital, Turin, Italy

**Keywords:** predictive factors, efficacy of radiofrequency ablation, radiofrequency ablation (RFA), benign thyroid nodules, volume reduction

## Abstract

**Purpose:**

To confirm the efficacy of ultrasound (US) guided radiofrequency ablation (RFA) in the treatment of benign thyroid nodules, we evaluated as primary outcome the technical efficacy and clinical success in a single center dataset. The secondary outcome was to find a correlation between nodules’ pre-treatment features and volume reduction rate (VRR) ≥75% at 12 months after RFA and during follow-up period.

**Methods:**

This retrospective study included 119 consecutive patients (99 females, 20 males, 51.5 ± 14.4 years) with benign thyroid nodules treated in our hospital between October 2014 and December 2018 with a mean follow-up of 26.8 months (range 3–48). Clinical and US features before and after RFA were evaluated by a US examination at 1, 3, 6, 12 months and annually thereafter up to 48 months.

**Results:**

The median pre-treatment volume was 22.4 ml; after RFA we observed a statistically significant volume reduction from the first month (11.7 ml) to the last follow-up (p < 0.001 for all follow-up times). The median VRR was 47.1, 55.3, 61.2, 67.6, 72.8, 71.3, and 62.9% at 1, 3, 6, 12, 24, 36, and 48 months of follow-up respectively, showing a progressive significant improvement up to 24 months (VRRs 1 *vs* 3 months, 3 *vs* 6 months and 6 *vs* 12 months p < 0.001, 12 *vs* 24 months p = 0.05) while no differences at 24 *vs* 36 and 36 *vs* 48 months were observed. Symptoms improved significantly (complete resolution 64.35%, partial resolution 35.65%), and neck circumference was reduced as compared to pre-treatment (p < 0.001). Lower pre-treatment neck circumference (37.5 *vs* 36.0 cm, p = 0.01) was a positive predictor of VRR ≥75% at 12 months. Macrocystic echostructure (HR 2.48, p 0.046) and pre-treatment volume >22.4 ml (HR 0.54, p 0.036) were found to be independent positive and negative predictors of VRR ≥75% respectively. One-month post RFA VRR ≥50% represented the best positive predictor of technical success.

**Conclusions:**

This study confirmed the efficacy of RFA in the treatment of benign thyroid nodules. In particular we show that by selecting macrocystic nodules smaller than 22.4 ml better long-term response can be achieved, which is predicted by an early shrinkage of the nodule.

## Introduction

Thyroid nodules are a relatively common clinical condition that affects up to 65% of the general population with large variability mostly originating from geographical heterogeneity and sensitivity of diagnostic methods (1). The incidence seems to be related to gender, insufficient iodine intake (2), and particularly, age (3). In most cases, these lesions are not symptomatic and, therefore, are diagnosed incidentally during instrumental examinations performed for some other reasons (4–6). Less frequently, thyroid nodules are diagnosed due to the presence of a palpable nodule in the cervical region or related symptomatology (*i.e.* compression trouble linked to the position and/or size) or thyroid hyperfunction (7, 8).

Although most thyroid nodules are benign, treatment could be required in the case of excessive size enlargement, compressive and/or cosmetic symptoms (9), or anxiety for the possibility of turning malignant (10, 11).

In these cases, the surgical approach represents the treatment to be preferred for thyroid nodules with compressive symptoms, even if it is known that neck surgery could lead to serious complications, *i.e.* hypocalcemia and dysphonia (12, 13).

For these reasons, in the last two decades, image-guided ablation procedures have been proposed. These procedures are minimally invasive and are also applicable to patients with contraindications to major surgery, or in patients who refuse it. The most consolidated alternative are percutaneous ethanol injection (PEI) (5, 14–16), laser ablation (LA) (17–20) and radiofrequency ablation (RFA) (21–26); more recently, micro-wave ablation (MW) and then high-intensity focused ultrasound (HIFU) have been proposed (27, 28).

In particular, RFA has been recommended as a treatment to be preferred for benign thyroid nodule by several guidelines (15, 29–32).

RFA of benign thyroid nodules have shown good results in volume reduction rate (VRR), ranging from 33 to 58% one month after treatment, and 51–85% up to six months, improving most of nodule-related clinical symptoms (21, 23, 24, 33–37).

However, the way of predicting the outcome of RFA treatments is still not well understood (38).

This study aimed to confirm the efficacy of RFA in the treatment of benign thyroid nodules in a single center dataset and to detect a significant correlation between pre-treatment features, including clinical and ultrasonographic features, and volume reduction rate (VRR) ≥75% at 12 months after the procedure and then during all follow-ups.

## Materials and Methods

This retrospective observational study included 119 consecutive patients referred to our Centre (Città della Salute e della Scienza University Hospital in Turin) for RFA of thyroid benign nodules from October 2014 to December 2018.

The inclusion criteria were: (a) patients aged 18 years or older; (b) confirmation of benignity (Tir2, SIAPEC-IAP) (39) or indeterminate lesions at low risk of malignancy (Tir3A) (39, 40) at two fine-needle aspiration cytology without echographic features suspicious for malignancy and normal level of serum calcitonin; (c) compressive or cosmetic symptoms in patients with refusal or ineligibility for surgery; (d) patient underwent one single-session RFA.

The exclusion criteria were: (a) malignant (Tir5) or suspicious of malignancy (Tir3b-Tir4) thyroid nodules, (b) pregnancy.

The Institutional Review Boards of our hospital approved this study, and patient consent was obtained in all cases.

### Pre-Treatment Assessment

#### Clinical Evaluation

We categorized symptom and cosmetic scores as defined in a previous consensus statement (41). Subjective compression symptoms were assessed by a visual analogue scale (grades 0–10) where 0 indicates the absence of compression-related disorders and 10 indicates the maximum tolerable discomfort; the cosmetic assessment was performed based on a four-point scale: 1 = absence of palpable mass; 2 = palpable but not visible mass; 3 = cosmetic problem during swallowing alone; 4 = easily identifiable visible mass.

Neck circumference, expressed in centimeters (cm) and measured by placing the tape measure in the middle of the neck of the patient, was evaluated.

#### Biochemical Evaluation

Laboratory tests included: thyroid stimulating hormone (TSH), serum free thyroxine (fT4), calcitonin, complete blood count, coagulation tests, dibucaine number and cholinesterase, hepatic and renal function. Additionally, all patients underwent baseline electrocardiogram and vocal cord function assessment performed by an otorhinolaryngologist before the ablation procedure.

#### Ultrasound Evaluation

Both transverse and longitudinal sonograms were obtained by real-time imaging of the thyroid nodules using an Esaote MyLab Twice real-time US system with a linear multifrequency (7–14 mHz) probe. Still and video clip sonographic images were evaluated by two board-certified radiologists (RG and SG) and two endocrinologists (RR and LP) with >10 years of experience.

The sonographic findings were analyzed based on the current guidelines (5, 15, 42) and reported as defined in a previous consensus statement (41).

Diameters (anteroposterior, transverse, and longitudinal) of each thyroid nodule were measured in centimeters (cm); the nodular volume, expressed in milliliters (ml), was calculated by the ultrasound machine on the basis of the diameters using the ellipsoid volume formula (length × width × depth × 0.524).

The nodular echostructure was classified as solid (≤10% of fluid component), microcystic (predominantly solid, 11–50% of fluid component), macrocystic (predominantly cystic, 51–90% of fluid component), cystic (>90% of fluid component), and spongiform **(**nodules containing multiple small cysts smaller than 5 mm interspersed within the solid tissue component for nearly all the volume).

The nodules echogenicity was classified as hypoechoic, isoechoic, or hyperechoic compared to the adjacent strap muscles of the neck. Regarding nodular shape, nodules were divided into regular or taller than wide. Nodular margins were categorized as smooth or irregular. Calcifications were reported as present or absent. Perinodular or intranodular vascularization was assessed by color and power Doppler examination; stiffness was evaluated by qualitative elastography (strain Elastosonography): the pressure is exerted freehand through the ultrasound transducer. An elastographic image (elasto-gram) is then produced, represented as a color-coded image superimposed on the image in mode B (43, 44); in our study, we used the classification proposed by Rago et al. (45). We defined the nodules with patterns 1 and 2 as soft; the nodules corresponding to pattern 3 were classified as intermediate elasticity and finally the nodules with patterns 4 and 5 were considered hard.

### RFA Procedure

Access to the procedures was carried out on a day hospital basis. RFA procedures were performed by an operator with an experience of >10 years (RG).

A single session of RFA was performed with the patient in a supine position with mild neck extension. Patients underwent treatment in a state of conscious sedation and were managed with 0.75% ropivacaine around the thyroid gland for puncture site anesthesia, always with ultrasound guidance.

We used an internally cooled electrode: 18 gauge, 7 or 10 cm length with a 10 mm active tip (*RFT(S) Tip/RFTP(S) RF Medical Co.Ltd.*) connected to a radiofrequency generator (Mygen M-3004).

A transisthmic approach method with the ‘moving shot technique’ (29, 46) was adopted; the insertion of the needle-electrode took place under freehand ultrasound guidance with a mid-lateral path, in such a way as to direct the flow of energy towards the lateral regions of the neck and away from areas at risk due to contiguity of thermal injury, such as the inferior laryngeal nerve and tracheoesophageal structures. The target nodule is ideally divided into several ablation units prior to the procedure. Starting from the deepest portion, the treatment is carried out unit after unit by moving the needle towards the most superficial portions. Due to the heat, necrosis is obtained and a hyperechoic area is formed on the tip of the needle-electrode; the generator shuts down power and impedance increases (47). The needle is then gradually brought back along the electrode axis in order to reach another tissue unit still to be treated. The procedure ends when all the ideal units of the nodule have been treated, which therefore appears completely hyperechoic.

The applied power and the actual time of treatment were recorded at the end of each session; the mean power used during the treatment was 55 Watt (W) and the treatment time about 15 min (49,500 J).

The procedures were monitored under the control of the B-mode ultrasound method in real time to assess the correct positioning of the needle-electrode within the lesion to be treated. A transient and complete hyperecogenicity of the target nodule, linked to heat-induced changes, represents the parameter that identifies the end of the procedure (48). Before removing the electrode, we performed an examination with Contrast Enhanced Ultrasound (CEUS), SonoVue (Bracco, Milan, Italy) to evaluate the extent of the necrosis area: ablation was considered complete when the total volume of the nodule appears not-vascularized. Once the needle-electrode was extracted, a new ultrasound evaluation was performed to exclude intra or extranodular complications.

The patients were observed for at least 3 h and were finally discharged, in some selected cases with the prescription of oral analgesic therapy.

### Follow-Up

Post-RFA, patients were followed up by US and clinical evaluations at 1, 3, 6 and 12 months after treatment and annually thereafter up to 48 months. In each follow-up, US examination, symptom and cosmetic score were evaluated while thyroid hormonal function was assessed every year. Thyroid nodule volume was assessed, and the volume reduction rate (VRR) of the treated nodule was calculated based on the formula: VRR = [(initial volume − final volume) ×100]/initial volume (26).

### Study Outcome

The primary outcome was the therapeutic efficacy in terms of volume reduction, VRR, and clinical success (41), defined as the ability of the treatment to resolve the condition itself (compression symptoms or cosmetic concerns); it was classified as complete (*i.e.*, complete resolution of presenting symptoms), partial (*i.e*., symptom improvement but still present), or absent (*i.e*., no symptom improvement) and by modification of cosmetic score.

The secondary outcome was to find a significant correlation between pre-treatment features of the nodules and technique efficacy, defined as a volume reduction ≥75% at 12 months after RFA and then during all follow-up period.

### Statistical Analyses

According to the descriptive statistics, continuous variables with normal distribution are expressed as mean ± standard deviation, while non-parametric data as median with interquartile range.

Statistical differences between continuous variables were evaluated with the Wilcoxon test for paired data or paired sample T-test.

To investigate the existence of an association between the technique efficacy of RFA (VRR ≥ 75%) and the clinical and pre-treatment ultrasound variables, a univariate analysis was conducted: Fisher’s χ2 test was used for binary and categorical variables, while, for the continuous variables, in consideration of the non-normal distribution of the data despite the good number of the sample under study, the Mann–Whitney U test was assessed.

Secondly, for the comparison between multiple groups, the Kruskal–Wallis H test was performed.

Finally, a multivariate logistic regression model was constructed to confirm the existence of independent variables.

A dynamic analysis was then carried out, considering for each subject the time, expressed in months, between the RFA treatment and a volume reduction rate ≥75%, considered as the technical success, or the time between treatment and the last follow-up, in case of a volumetric reduction <75%. It was possible to evaluate the cumulative incidence of success (VRR ≥ 75%) by constructing the Kaplan–Meier curves, for a maximum follow-up period of 48 months; by using the Log Rank test, the Kaplan–Meier curves were then compared to evaluate the existence of possible predictors. For continuous variables, this test was performed by dividing the subjects into two categories based on the median of distribution of the variable itself.

Finally, a multivariate Cox regression model was constructed to confirm the presence of independent predictors.

The results were considered statistically significant if the p-value was less than 0.05.

All statistical analyses were performed with STATA IC 10 (STATACORP, LP, Texas, USA) analytic software.

## Results

### Demographic and Sonographic Characteristics of Thyroid Nodules

In this study, 99 out of 119 patients were females (83.20%); the mean age at treatment was 51.5 ± 14.4 years with a mean follow-up of 26.8 months (range 3– 48) after RFA. All patients had normal TSH value. As regards the echostructural ultrasound pattern, out of a total of 119 nodules, 29 (19.33%) were solid, 62 (52.1%) microcystic, nine (7.56%) macrocystic, and finally 11 (9.24%) spongiform. No cases of pure cystic nodules were treated. Regarding the nodules with the first cytological result of TIR3A, they were TIR2 at the second fine-needle aspiration cytology in four out of five cases; only one case was confirmed as TIR3A, GALECTIN 3 negative for immnocytochemistry. In this case, the intermediate ultrasound risk nodule (15) with a size >3 cm created tracheal compression. The patient refused surgery. The baseline clinical and ultrasound characteristics of the patient and nodules are summarized in **Tables 1** and **2** respectively.

**Table 1:** Pre-treatment clinical features of 119 thyroid RFA-treated nodules.

Clinical Features		Total n = 119 (%)
Thyroid function	Euthyroidism	100 (83.00)
	Hypothyroidism	16 (13.50)
	Hyperthyroidism	3 (2.50)
Citology	Tir2	114 (95.80)
	Tir3A	5 (4.20)
Compressive symptoms (0–10)	0	3 (2.52)
	1	2 (1.68)
	2	14 (11.76)
	3	13 (10.92)
	4	14 (11.76)
	5	35 (29.41)
	6	19 (15.97)
	7	7 (5.88)
	8	9 (7.56)
	9	2 (1.68)
	10	1 (0.84)
Cosmetic score (1–4)	1	1 (0.84)
	2	2 (1.68)
	3	2 (1.68)
	4	114 (95.80)
**Clinical Features**	**Mean ± DS**	**Median** **(interquartile range)**
Neck circumference (cm)	37.59 ± 3.57	37 (5.00)
Volume (ml)	25.25 ± 16.81	22.4 (20.70)

**Table 2:** Ultrasonographic features of 119 thyroid RFA-treated nodules.

Ultrasound Features		Total n = 119 (%)
Structure	Solid	29 (19.33)
	Microcystic	62 (52.10)
	Macrocystic	9 (7.56)
	Spongiform	11 (9.24)
Echogenicity	Anechoic	0 (0.00)
	Isoechoic	72 (60.50)
	Hypoechoic	47 (39.50)
	Markedly Hypoechoic	0 (0.00)
Margins	Regular	116 (97.48)
	Irregular	3 (2.52)
Calcifications	Absent	80 (67.23)
	Present	29 (32.77)
Shape	Regular	119 (100)
	Taller than wide	0 (0.00)
Vascularization	Perinodular	29 (24.37)
	Endonodular	0 (0.00)
	Peri-Endonodular	88 (73.95)
	Unknown	2 (1.68)
Elastosonography	Soft	42 (35.29)
	Intermediate	52 (43.70)
	Hard	3 (2.52)
	Unknown	22 (18.49)
Volume (mL)	≤ 10	21 (17.65)
	11-30	60 (50.42)
	>30	38 (31.93)

### Volume and VRR

**Figure 1:**
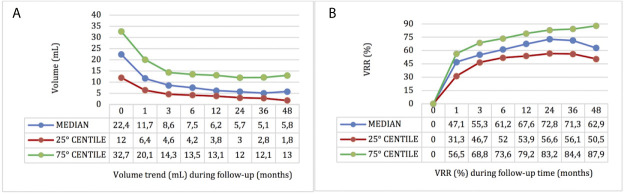
Nodules’ volume **(A)** and VRR **(B)** by time after RFA.

The median pre-treatment volume was 22.4 ml; after RFA we observed a statistically significant volume reduction from the first month (11.7 ml) to the last follow-up (p < 0.001 for all follow-up times). The median VRR was 47.10% (range 31.30–56.50), 55.30% (range 46.70–68.80), 61.20% (range 52.0–73.60), 67.60% (range 53.90–79.20), 72.80% (range 56.60–83.20), 71.30% (range 56.10–84.40), 62.90% (range 50.50–87.90), at 1, 3, 6, 12, 24, 36, and 48 months of follow-up respectively, showing a progressive significant improvement up to 24 months of follow-up (VRRs 1 *vs* 3 months, 3 *vs* 6 months and 6 *vs* 12 months p < 0.001, 12 *vs* 24 months p = 0.05), while no significant differences between VVRs at 24 *vs* 36 months and 36 *vs* 48 months were observed (**Figure 1**).

**Figure 2:**
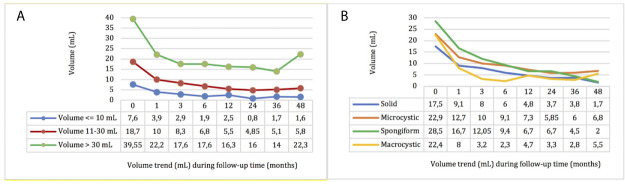
Nodules’ volume by time after RFA stratified by pre-treatment volume **(A)** and echostructure **(B)**.

**Figure 3:**
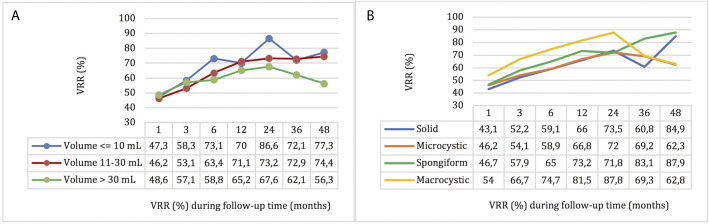
Nodules’ VRR by time after RFA stratified by pre-treatment volume **(A)** and echostructure **(B)**.

Stratifying the study sample into three categories based on the pre-treatment volume (41) (nodules ≤ 10, 10–30, and >30 ml), a significant volume reduction was confirmed for each category (p <0.001) (**Figure 2**). Furthermore, a significant improvement in VRR was confirmed for each category (p < 0.001), and in the comparison between the three groups, nodules with volume ≤10 ml had a higher VRR although in a non-statistically significant way (**Figure 3**).

These results were also observed dividing the study sample into four categories by pre-treatment echostructure (41); a significant and progressive reduction in volume (p <0.001) and improvement in VRR (p <0.001) were confirmed for each echo pattern (**Figure 2**) and the nodules with macrocystic and spongiform echostructure showed a higher VRR compared to the other groups, but not statistically significance (**Figure 3**).

### Clinical Success

Compressive symptoms (complete resolution 64.35%, partial resolution 35.65%) and cosmetic score improved significantly (p <0.001); a statistically significant reduction in neck circumference was obtained at 6, 12, and 24 months post-treatment (p <0.001). The mean pre-treatment neck circumference was 37.59 ± 3.57 cm and decreased to 36.02 ± 3.43, 35.94 ± 3.02, and 35.79 ± 2.92 cm respectively at 6, 12, and 24 months post-treatment.

### Technical Success (VRR ≥75% at 12 Months) of Predictors’ Analysis

At the end of the global follow-up, 108 out of 119 patients reached a VRR ≥50%.

The patients were divided into two groups in relation to the percentage of volumetric reduction obtained 12 months after treatment: BR (Best Responders) group in case of VRR ≥75% (36 cases) and MR (Mild Responders) group in case of VRR <75% (75 cases); subsequently, variables associated with VRR (predictors) were analyzed.

The two groups did not show gender (p = 0.645) or age (p = 0.731) differences regardless of the cytological result.

Regarding neck circumference before treatment, the BR group showed significantly lower values (p = 0.012) than the MR group.

In terms of volume, the BR group had lower nodular volumes than the MR group, with a difference close to statistical significance (p = 0.063).

Analyzing nodular echostructure, the microcystic (p = 0.037) and macrocystic (p = 0.022) nodules were associated with a VRR ≥75% at 12 months.

Finally, we analyzed as a possible positive predictor of response a VRR ≥50% at 1 month (VRR1m), considered the minimum percentage of RFA success by recent literature (41); this binary parameter was able to differentiate the two groups (p < 0.001), and specifically, the BR group presented a greater VRR1m than the MR group (56.30 *vs* 40.30%).

At multivariate logistic regression analysis, only VRR1m ≥50% was confirmed as an independent predictor of a VRR ≥75% at 12 months after treatment (p = 0.001; OR: 6.91; CI: 2.23–21.45).

### Time-Dependent Predictors’ Dynamic Analysis

The cumulative incidence of the technical success curve showed that most of the RFA technical success was achieved within 12 months (36%) with a progressive and clear improvement up to 24 months (48%). After 24 months, although there was a slight effectiveness improvement, it was significantly lower than the effectiveness obtained within 24 months.

By comparing the Kaplan–Meier curves, we observe that gender, older age (over the median value, 50 year), and larger neck circumference (over the median value, 37.5 mm) do not significantly affect the final outcome.

**Figure 4:**
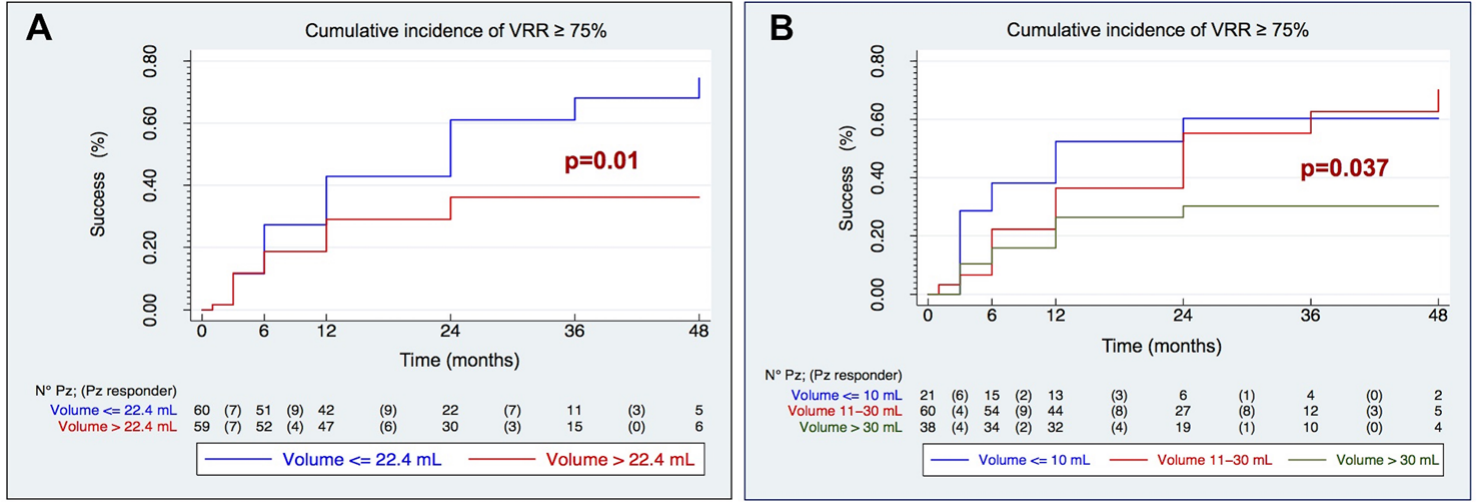
Kaplan–Meier curves of cumulative incidence of technical success (VRR ≥ 75%) by median volume **(A)** and three volume categories **(B)**.

The pre-treatment volume is found to be a good predictor of treatment efficacy (p = 0.01). The group of patients with a nodular volume ≤22.4 ml (median) responded to the treatment better than the others; on the contrary, a pre-treatment volume >22.4 ml represented a negative predictor of success with an overall efficacy that never exceeds 40%, **Figure 4**.

The pre-treatment volume was confirmed as a good predictor of technical success even when stratified into three categories, as proposed by the recent literature (p = 0.037), **Figure 4**.

In fact, a clear divergence of the three curves is observed; in particular, the effectiveness of the treatment is found very low (≤30%) in nodules >30 ml, while the technical success of RFA is rapidly increasing in nodes <30 ml, especially if ≤10 ml (52% at 12 months, 60% at 24 months).

**Figure 5:**
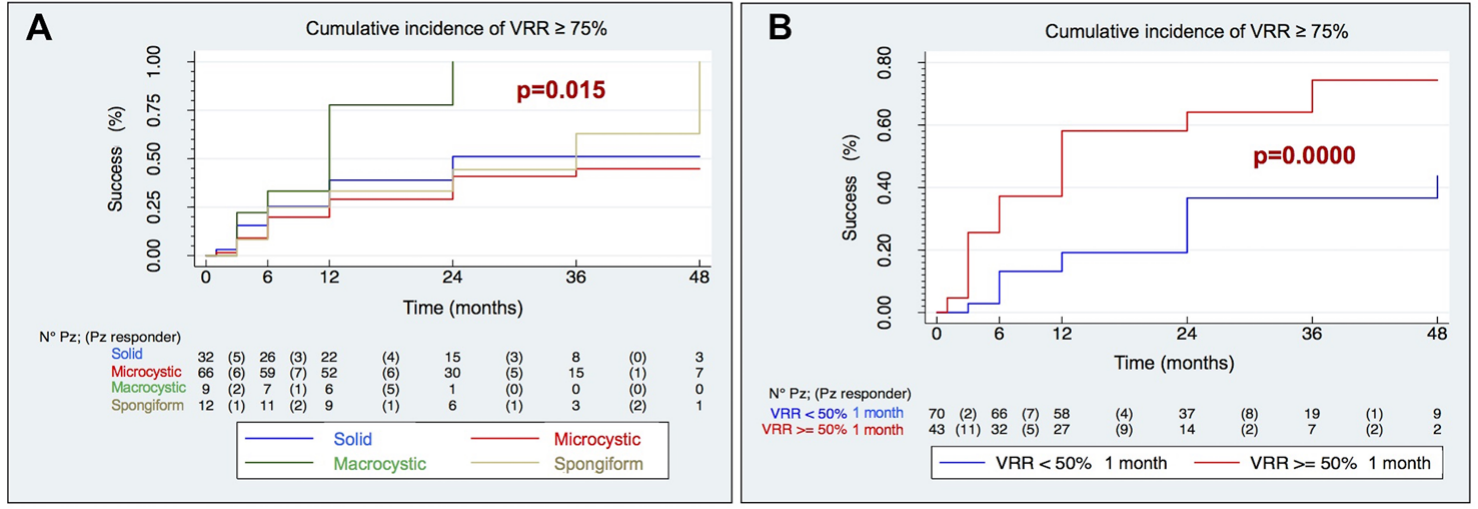
Kaplan–Meier curves of cumulative incidence of technical success (VRR ≥ 75%) by echostructure **(A)** and VRR at 1 month **(B)**.

The comparison of the Kaplan–Meier curves shows that pre-treatment echostructural patterns are able to separate the curves with good reliability (p = 0.015). In particular, it shows how the macrocystic echostructure positively modifies the outcome of the treatment with an efficacy of over 75% at 12 months and almost total at 24 months, **Figure 5**.

Finally, taking into consideration the post-treatment variables, Kaplan–Meier curves highlight that an early volumetric reduction, as evidenced by a VRR ≥50% at 1 month after treatment, was an excellent predictor of the achievement of a VRR ≥75% during the whole follow-up (p = 0.0000), **Figure 5**.

At multivariate Cox regression model (p = 0.037, χ^2^: 13.41), macrocystic echostructure (HR 2.48, IC: 1.02–6.07 p 0.046) and pre-treatment volume >22.4 ml (HR 0.54, IC: 0.31–0.96, p 0.036) were found independent positive and negative predictors of VRR ≥75% respectively.

One-month post RFA VRR>50% represented the best positive predictor of technical success (HR: 2.48; CI: 1.40–4.39, p = 0.002).

## Discussion

RFA treatment of benign thyroid nodules aims at obtaining a sufficient reduction of nodule volume for the regression of the compressive symptoms and cosmetic disorder, keeping it as stable as possible over time (15, 29, 30).

Our retrospective study confirmed the efficacy and safety of RF ablation in a single large cohort of patients. Particularly, the nodular volume showed a notable reduction as early as 1 month post-RFA, with a median VRR of 47.10% at 1 month of follow-up and 72.80% at 24 months, according to the literature (49–52).

RFA treatment of benign nodules has been introduced into clinical practice for a few years, so it still has a time-limited follow-up (51, 53, 54). To this purpose, this study is expected to provide additional information on the long-term result (up to 48 months) in terms of volume reduction (VRR) and the possible correlation between pre-treatment features and volume reduction rate.

First of all, our results (VRR at 3 months: 55.30%; VRR at 6 months: 61.20%) agree with some studies reporting that the achievement of a VRR ≥50% is detectable already at 6 months follow-up (52, 55). Then, in the literature, the technical success is usually identified in a VRR ≥50% at 12 months (41) which, at best, was achieved by 97.8% of patients (50). In our study, the achievement of RFA technical success, defined as a VRR ≥75%, was found in 36% of cases, with progressive improvement up to 24 months follow-up (48%) and beyond, although less significant, achieving a VRR ≥75% overall in the majority of treated nodules (57%).

Furthermore, according to our results, clinical success of the treatment was complete in most cases within 12 months post-RFA (64.35%); in the other cases the reduction was partial (35.65%), indicating that none of the patients undergoing RFA had an unsuccessful treatment, as highlighted by other authors (51, 52, 55). However, evaluation of symptoms and clinical success deriving from the volumetric reduction is subjectively expressed, and the results are not completely reliable as indicators of the success of the technique.

To date in the literature, some characteristics (47, 54), such as a spongiform echostructure, a liquid component, a smaller initial pre-treatment volume, an intense peripheral and intranodal pattern vascularity of the nodule, have been found positively correlated with a better result in terms of volumetric reduction, but no predictive pre-treatment factors of RFA success have been identified definitely. For this reason, we conducted a specific analysis to identify the differences between clinical and ultrasound parameters in best responder and mild responder patients.

Between the two groups, the indicative factors of a better technical success proved to be:

- the echostructural pattern: in our series, the best technical success was observed in macrocystic nodules (p = 0.015), in agreement with the literature (47, 56). The possible explanation for this is that, as is known, the heating of a tissue with a large fluid component, (be it colloid or blood) produces a greater amount of vapor and a higher temperature which favors the thermocoagulation process in the treated nodule;
- the pre-treatment neck circumference, lower in the best responder group, indicates that a larger neck is associated with a worse response to the treatment. This data, never considered in the literature, would deserve further prospective studies in our view. Since there are no other systems beyond the ultrasound to assess the goodness of the result, we have tried to insert a linear dimensional parameter such as the circumference of the neck in its maximum diameter. This parameter, never considered in the literature, could offer further important prospective studies. Certainly, this parameter is associated with some confounding factors, as weight changes; however, the measurement of the neck circumference pre-treatment and during the follow-up allows us to hypothesize that the volumetric reduction of a nodule in the thyroid induces a remodeling of the anatomical structures contained in the neck.
- the pre-treatment nodular volume: in best responding patients, the nodules with volume ≤22.4 ml had a substantially better response (p = 0.01). This result reinforces the hypothesis that the greater volume reduction in nodules with lower basal volume is probably due to reduced energy deposition during RFA within large ones, which therefore results in a lower response (32, 51).

In addition, in our cohort, we have pointed out that VRR1m is a good predictor of technical success (reaching 50% in 56.30% of responding patients; p = 0.0000); however, it should be emphasized that this parameter, not mentioned in the literature, although reliable, can only be evaluated after treatment and, therefore, does not fall within the definition of predictor of technical success.

Our study also highlights a progressive improvement in technical success up to 24 months (48%) which remains stable in the following months without however showing further significant volume reductions. This data allows us to hypothesize therefore a stability of the effectiveness of the technique over a long period.

In clinical practice of management of symptomatic thyroid nodules, these data could strengthen the indication of using RFA on nodules with small volumes, without waiting for a volumetric increase that could make this treatment less effective.

In addition, we observe that the measurement of the neck circumference, despite possible confounding, combined with the ultrasound evaluation, could provide important information on the outcome of the procedure.

Finally, the strong correlation detected between VRR1m and technical success shows the importance of monitoring the nodular volumetric reduction during the first month post-RFA. This parameter could be considered as an important indicator of a successful outcome of the procedure (VRR1m >50%), or, on the contrary, it can hypothesize a poor success of the procedure, suggesting for example, to plan a second RFA or surgery treatment in accordance with the patient’s opinion.

The major limitation is the retrospective nature of the study, which however, has the strength of having been conducted in a single center by the same personnel, thus lowering the risk of interobserver bias.

With the limit of a retrospective study, regarding the neck circumference as a factor to control in the follow-up, we have reported only data on cases of changes in body weight, but we have not adjusted the neck circumference for this parameter. However, looking at these cases, we can state that in this study the reduction in neck circumference occurred regardless of the change in body weight.

Unfortunately, in this retrospective study we did not collect data on thyroid autoantibodies. Further prospective studies should consider the role of thyroiditis in structural changes of the nodule after RFA.

An interesting future development could be the evaluation of the energy delivered during RFA procedures in order to identify energy values to be used for improving the final outcome.

## Conclusion

Based on current findings, the selection of nodules for pre-treatment allows for better long-term responses, especially for nodules with a lower pre-treatment volume (≤22.4 ml) and/or a macrocystic echostructure; early shrinkage of the nodule, as observed by a VRR1m ≥50% at one-month follow-up, is shown to be a good predictor of positive RFA responses. Therefore, these factors represent the best positive predictors of the radiofrequency thermal ablation technique on benign thyroid nodules.

In conclusion, these parameters should always be evaluated before considering any treatment with RFA to estimate the probability of success or failure of the therapeutic method that is targeted for each case. This result has important implications from a clinical point of view: (i) patients can be made more confident on the resolution of reported symptoms, and (ii) it provides a valid alternative to the surgical approach while permitting to gain relevant information on the need of repeated treatment sessions, if these factors are absent.

## Data Availability Statement

The raw data supporting the conclusions of this article will be made available by the authors, without undue reservation.

## Ethics Statement

The studies involving human participants were reviewed and approved by Comitato Etico Interaziendale A.O.U. Città della Salute e della Scienza di Torino. The patients/participants provided their written informed consent to participate in this study.

## Author Contributions

All authors contributed to the study conception and design. Material preparation and data collection were performed by AB, RR, LP, SG, AF, and AC. Data analysis and table designing were performed by AB and MM. The first draft of the manuscript was written by AB and all authors commented on previous versions of the manuscript. EG, MP, MM, and RG verified the analytical methods and supervised the manuscript drafting. All authors contributed to the article and approved the submitted version.

## Conflict of Interest

The authors declare that the research was conducted in the absence of any commercial or financial relationships that could be construed as a potential conflict of interest.
